# Bisphosphonate Use and Fractures in Adults with Hypophosphatasia

**DOI:** 10.1002/jbm4.10223

**Published:** 2019-08-26

**Authors:** Kate Rassie, Michael Dray, Toshimi Michigami, Tim Cundy

**Affiliations:** ^1^ Department of Endocrinology Greenlane Clinical Centre Auckland New Zealand; ^2^ Department of Pathology Waikato Hospital Hamilton New Zealand; ^3^ Department of Bone & Mineral Research Osaka Women's & Children's Hospital Isumi Japan; ^4^ Department of Medicine, Faculty of Medical & Health Sciences University of Auckland Auckland New Zealand

**Keywords:** *ALPL*, ATYPICAL FEMORAL FRACTURES, GENE MUTATIONS, HYPOPHOSPHATASIA, OSTEOMALACIA, OSTEOPOROSIS

## Abstract

Adults with hypophosphatasia (HPP) may suffer femoral fractures resembling the atypical femoral fractures that can occur with long‐term bisphosphonate treatment, and there is an emerging consensus that bisphosphonates should not be used in adults with HPP and low bone mass. However, the spectrum of HPP in adults is wide: ranging from the severely affected—who commonly have osteomalacia—through to the minimally affected. The former typically have biallelic and the latter, heterozygous *ALPL* mutations. We have reviewed reports of fractures in adults with genetically proven HPP which suggest that the risk of fracture is at least 200‐fold greater in those with biallelic mutations. We also discuss two cases of postmenopausal women with heterozygous *ALPL* mutations. One had fractures and severe osteoporosis, but histology revealed no evidence of osteomalacia. The second had taken alendronate for 8 years, but despite profound suppression of bone turnover, histology again revealed no evidence of osteomalacia. The management of adults with HPP who have coexisting osteoporosis is challenging. More data are clearly needed, but we suggest that the risks of bisphosphonate therapy may be relatively low in patients who have heterozygous mutations and no histological evidence of osteomalacia. © 2019 The Authors. *JBMR Plus* published by Wiley Periodicals, Inc. on behalf of American Society for Bone and Mineral Research © 2019 The Authors. *JBMR Plus* published by Wiley Periodicals, Inc. on behalf of American Society for Bone and Mineral Research.

## Introduction

Hypophosphatasia (HPP) is an inborn error of metabolism, caused by mutation of the gene *ALPL* that encodes the enzyme tissue non‐specific alkaline phosphatase. Alkaline phosphatase (ALP) has a critical role in facilitating the mineralization of osteoid. Its main substrate in bone is pyrophosphate, a potent natural inhibitor of mineralization. ALP cleaves pyrophosphate into its two phosphate moieties, which then become available to the mineralization process. In severe hypophosphatasia, excess pyrophosphate accumulation at the mineralization surface causes defective mineralization, and bone histology shows osteomalacia. In lethal perinatal cases there may be almost no mineralization of bone, and cases recognized in infancy or childhood have rickets. Milder forms are typically diagnosed in adulthood after sustaining fractures, although a history of early loss of primary dentition (odontohypophosphatasia) is common.^1^ Other adults may be asymptomatic, with the diagnosis made incidentally; it is probable that many adults with mild HPP never come to clinical attention. Although other proteins and molecular pathways may be important in modulating the clinical phenotype, the clinical severity of HPP is related primarily to the degree of residual ALP activity. This in turn is related to the nature of the mutation (whether or not critical functional domains of the enzyme are affected) and whether one or both *ALPL* alleles carry mutations.

Bisphosphonates, which are now extensively used in the treatment of osteoporosis, are analogues of pyrophosphate. They were originally developed for industrial purposes to prevent calcification. The prototype bisphosphonate used in humans, etidronate, can cause osteomalacia when taken in high doses.^2^ Although the second and third generation bisphosphonates in current clinical use do not have this effect, a number of recent case reports have documented so‐called atypical femoral fractures occurring in adults with HPP who have been treated with bisphosphonates.^3^, ^4^, ^5^, ^6^, ^7^, ^8^ Review articles and expert opinion now caution against their use in individuals with HPP.^9^, ^10^ However, there are a number of important questions that remain unanswered. Although “atypical” fractures affecting the proximal/lateral femoral sites differ from those classically described in osteomalacia (where the femoral neck is a common site of fracture), they are also well documented to occur in HPP patients who have not been treated with bisphosphonates. To what extent does bisphosphonate treatment add to the risk? What is actually happening in bone in such cases? Are these fractures related to the induction of osteomalacia, or to the suppression of bone turnover by bisphosphonates^9^, ^11^? Is the fracture risk the same for individuals with a single heterozygous mutation as it is for those with bi‐allelic mutations (homozygous or compound heterozygous), who generally have less residual ALP activity?

In this article, we present two cases illustrating the dilemma of managing osteoporosis in adults with heterozygous *ALPL* mutations, and offer some pathological insights based on bone biopsy findings. We also review published cases of atypical fractures in individuals with HPP and confirmed *ALPL* mutations.

## Patients and Methods

### Case histories

*Case 1*. A 62‐year‐old woman presented with sacral and multiple vertebral fractures after having fallen from a ladder. A bone density scan confirmed osteoporosis with T‐scores of −5.2 at the lumbar spine, −3.5 at the femoral neck, −3.3 at the total femur, and −2.6 at the total radius sites. Apart from low body weight and post‐menopausal status, no other risk factors for osteoporosis were identified. Family history was unremarkable. She had normal plasma calcium, phosphate, PTH, and 25‐OH vitamin D levels. However, she was noted to have low serum ALP of 35 U/L, raising the possibility of HPP. Her pyridoxal 5′‐phosphate level was elevated, supporting the diagnosis (Table 1). *ALPL* gene sequencing confirmed heterozygosity for a deletion–insertion mutation c.650delT insCTAA (p.217 delVal insAlaLys) in exon 7 of *ALPL*, previously reported in a patient with lethal perinatal HPP and compound heterozygous *ALPL* mutations.^12^

**Table 1:** Clinical Details and Laboratory Findings

Case	1	2
Gender/age (years)	F/62	F/61
BMI (kg/m^2^)	17.9	18.7
Fractures	vertebral, sacral	none
BMD (lumbar spine: *T*‐score)	−5.2	−2.0
Biochemistry		
Pyridoxal 5'phosphate (nmol/L) Normal range 35 to 107	194	252
ALP (μ/L) before bisphosphonate treatment Normal range 40 to 120	35	28
ALP (μ/L) on bisphosphonate treatment Normal range 40 to 120	21	10
P1NP (μg/L) before bisphosphonate treatment Normal range 10 to 110	60	NA
P1NP (μg/L) on bisphosphonate treatment Normal range 20 to 110	NA	5
Quantitative bone histology		
Trabecular bone volume (%) Normal range 22.5 ± 3.5	13.5	12.8
Osteoid volume (%) Normal range 1.9 ± 0.4	1.6	0.8
Osteoid surface (%) Normal range 19.3 ± 3.0	13.3	11.4
*ALPL* mutation		
cDNA	650 delT insCTAA	814C > T
Protein	217 delV insAK	R272C

ALP = alkaline phosphatase; NA = not available.

We took a trans‐iliac bone biopsy to determine if there was evidence of osteomalacia. The biopsy showed the trabecular bone to be osteopenic, with reduced bone volume and slender trabeculae. The cortex was of normal thickness, with a mild increase in porosity. There was no evidence of hyperosteoidosis (Table 1).

*Case 2*. A 53‐year‐old woman with no history of fracture had a bone density scan 4 years after her menopause. This showed osteopenia and she was prescribed alendronate 70 mg weekly. After 8 years of treatment she was noted to have a very low ALP level of 10 to 15 μ/L, and was referred for further investigation. Plasma calcium, phosphate and PTH levels were normal; there was no relevant family history. Her bone turnover was low, as judged by a P1NP level of 5 μg/L. ALP measurements made before she started alendronate were retrieved: These had been low at 24 to 28 μ/L, but their significance had been overlooked. Her pyridoxal 5′‐phosphate level was elevated, supporting a diagnosis of HPP. Sequencing confirmed heterozygosity for a missense mutation c.814C>T (p.Arg272Cys) in exon 8 of the *ALPL* gene previously reported in a patient with severe perinatal HPP and compound heterozygous *ALPL* mutations.^13^ A transiliac bone biopsy was taken because of concern that the bisphosphonate treatment may have induced a mineralization defect. The biopsy showed the trabecular bone to be osteopenic with reduced bone volume, but no evidence of hyperosteoidosis (Table 1).

### Laboratory methods

Genomic DNA was extracted from peripheral blood leucocytes. Genomic PCR was performed using the primers as previously described.^14^ The amplified fragments were gel‐purified and directly sequenced. The nucleotide and amino acid numbers were designated relative to the initiation of cDNA and the translational start site, respectively.

Transiliac bone biopsies were taken under local anaesthesia and light sedation, using an 8‐mm trephine. After dehydration in ascending concentrations of ethanol, the undecalcified samples were embedded in methylmethacrylate, and 5‐mm‐thick sections were cut using a microtome, deplasticized, and the resin removed before staining. Quantitative histomorphometry was undertaken on von Kossa or Goldner trichrome‐stained and unstained tetracycline fluorescence sections using the OsteoMeasure histomorphometry system (Osteometrics, Atlanta, GA, USA).

### Literature review

Table 2 summarizes data from published case reports and series that have described femoral fractures in adults with HPP confirmed by genetic analysis. The Table also includes data on 3 patients who had multiple fractures (but not including a femoral fracture).^3^, ^4^, ^5^, ^6^, ^7^, ^8^, ^14^, ^15^, ^16^, ^17^, ^18^, ^19^, ^20^, ^21^, ^22^, ^23^, ^24^ Fifteen of the 34 subjects had been exposed to bisphosphonates. Twenty‐five (74%) subjects were considered to have clinical features suggestive of HPP and 23 (67%) had bi‐allelic *ALPL* mutations. Bone histology was reported in only 3 subjects, all of whom had bi‐allelic mutations and osteomalacia.^14^, ^18^, ^24^

**Table 2:** Published Cases of Atypical Femoral Fractures in Adults with Hypophosphatasia (HPP) and Proven *ALPL* Mutation

References	Age/sex	Unilateral or bilateral femoral fractures	Bisphosphonate exposure	*ALPL* mutations	Other clinical features of HPP	Comment
Sutton *et al*. (2012)^3^	55/F	Bilateral	ALN/ZOL – 4 year	Heterozygous	No	
Sum *et al*. (2013)^4^	66/F	NS	NS	Heterozygous	No	
Cundy *et al*. (2015)^14^	52/M	Other fractures	ALN – 2 year	Comp heterozygous	PLPD	Renal failure; osteomalacia
Genest and Seefried (2018)^5^	57/F	Bilateral	ALN or PAM or ZOL 1 to 11 year (mean 5.3 year)	Homozygous	Yes	
	85/F	Bilateral		Comp heterozygous	Yes	
	71/F	Bilateral		Comp heterozygous	Yes	
	62/F	Bilateral		Comp heterozygous	Yes	
	76/F	Bilateral		Comp heterozygous	Yes	
	55/M	Bilateral		Comp heterozygous	Yes	
	73/F	Bilateral		Comp heterozygous	Yes	
Righetti *et al*. (2018)^6^	67/F	Bilateral	ALN – 10 year	Heterozygous	PLPD	Also had steroid exposure
Lefever *et al*. (2018)^7^	36/F	Unilateral	PAM ‐ age 16 to 23	Homozygous	Yes	
	69/F	Unilateral	RIS – 6 year	Heterozygous	Yes	Denosumab 1½ year after RIS
Camacho *et al*. (2018)^15^	75/F	Other fractures	IBN – 7 m age 68	Heterozygous	No	Also had steroid exposure
Peris *et al*. (2019)^8^	67/F	Unilateral	ALN – 8 year	Heterozygous	No	
Khandwala *et al*. (2006)^16^	64/F	Unilateral	None	Heterozygous	Yes	
Whyte *et al*. (2007)^17^	56/F	Bilateral		Heterozygous	PLPD	
Gagnon *et al*. (2010)^18^	53/F	Bilateral		Comp heterozygous	No	Osteomalacia
Schalin‐Jäntti *et al*. (2010)^19^	54/F	Bilateral		Comp heterozygous	Yes	
	64/F	Bilateral		Comp heterozygous	NS	
Laroche (2012)^20^	43/F	Bilateral		Heterozygous	Yes	
Maman *et al*. (2016)^21^	51/F	Bilateral		Comp heterozygous	PLPD	
Braunstein, (2016)^22^	43/M	Others		Heterozygous	No	
Lawrence *et al*. (2017)^24^	55/F	Bilateral		Comp heterozygous	No	Osteomalacia
Camacho *et al*. (2018)^15^	53/F	Other fractures		Heterozygous	No	
Klidaras *et al*. (2018)^23^	41/F	Bilateral		Comp heterozygous	Yes	Asfotase alfa‐treated
	61/M	Bilateral		Comp heterozygous	No	Asfotase alfa‐treated
Genest and Seefried (2018)^5^	55/F	Bilateral		Comp heterozygous	Yes	
	39/M	Unilateral		Comp heterozygous	Yes	
	46/F	Bilateral		Comp heterozygous	Yes	
	51/F	Bilateral		Comp heterozygous	Yes	
	45/F	Unilateral		Comp heterozygous	Yes	
	43/F	Bilateral		Comp heterozygous	Yes	
	50/F	Bilateral		Comp heterozygous	Yes	

ALN = alendronate; PAM = pamidronate; RIS = risedronate; IBN = ibandronate; ZOL = zoledronate; PLPD = premature loss of primary dentition; NS = not stated.

## Discussion

The fractures sustained by the woman described in case 1 together with severe osteoporosis on bone density scanning would ordinarily be an indication for bisphosphonate treatment. Should the discovery that she was a carrier of a single *ALPL* mutation mean that bisphosphonate therapy be withheld because of concern that she might be susceptible to developing atypical femoral fractures? In attempting to answer this question, there are several points that need to be considered.

First, three of the six heterozygous mutation carriers with femoral fractures in our review of the existing literature (Table 2) had 6 or more years' of continuous bisphosphonate exposure. As the risk of bisphosphonate‐associated atypical femoral fracture increases with duration of use,^25^ their risk may well have been increased irrespective of their *ALPL* mutation status.

Second, though femoral fractures are common in adults with HPP^5^, ^26^ our literature survey (Table 2) found that only a third of cases were reported in people with heterozygous *ALPL* mutations. Between 1 in 80 and 1 in 100 people carry single *ALPL* mutations, meaning that between 1 in 6400 and 1 in 10,000 carry bi‐allelic mutations. This suggests that the risk of atypical fractures is at least two orders of magnitude greater in people with bi‐allelic mutations than in single mutation carriers. It is also interesting to note that the majority (74%) of the individuals with bi‐allelic mutations had, in the opinion of the authors surveyed, phenotypic features of HPP (in addition to low plasma ALP activity), suggesting that they could have been identified clinically. These phenotypic features of adult HPP include early loss of permanent dentition, bone pain, and recurrent and poorly healing fractures^27^ as well as skeletal deformities, osteomalacia, chondrocalcinosis, and pyrophosphate arthropathy. In some cases, a careful history and examination in an individual with bi‐allelic mutations will suggest a diagnosis of paediatric HPP that has gone unrecognized (suggestive symptoms include early loss of primary dentition, rickets, gait abnormalities). Such symptoms are less likely to be present in the patients heterozygous for HPP, the majority of whom have no clinical features of the disease.

Third, data on histology are very limited, but in 3 subjects with bi‐allelic mutations there was clear evidence of osteomalacia.^14^, ^18^, ^24^ One could then hypothesize that those with osteomalacia are at the greatest risk of fracture, and that those with bi‐allelic mutations are more likely to have osteomalacia. Published studies support the view that not all adults diagnosed with HPP have osteomalacia. Fallon and colleagues found osteomalacia only in adults who had severe skeletal symptoms, but not in those with milder disease (characterized by odontohypophosphatasia but no other skeletal symptoms).^28^ Bersketh and colleagues found osteomalacia in only two of four minimally symptomatic adults with HPP.^29^ In contrast, Barvencik and colleagues found osteomalacia was present in adults with HPP who had skeletal symptoms.^30^

In a previously published case of a man carrying biallelic mutations who had incidental renal failure, we observed a marked increase in fracture rate after alendronate treatment.^14^ In this case, it was probable that the combination of bisphosphonate and renal failure (the latter impairing clearance of the drug) changed a state of moderate osteomalacia with the capacity to repair defects into a severe, “frozen” low turnover osteomalacia state with accumulation of pyrophosphate on mineralizing surfaces and almost no new bone formation.^14^

But can bisphosphonate therapy induce osteomalacia in an asymptomatic carrier of a single *ALPL* mutation? The evidence on this point is even more limited, but in case 2 described here, 8 years' treatment with alendronate, though profoundly suppressing bone turnover, did not cause a mineralization defect. Of note, aminobisphosphonate therapy in normal post‐menopausal women with osteoporosis has no effect on the hydrolysis of phosphate esters by ALP.^31^

Fourth, BMD is normally distributed in adults with HPP, so low bone mass, as measured by densitometry, is not a characteristic feature.^32^ However, it is those with low bone density (with or without fractures) who are most likely to come to medical attention. This may explain the preponderance of post‐menopausal women included in the studies listed in Table 2. Thus in some cases, mild or asymptomatic HPP will be a diagnosis incidental to that of post‐menopausal osteoporosis. Should heterozygous *ALPL* mutation carriers with osteoporosis be treated differently, then, to other individuals with osteoporosis? Possibly not—but more data are needed before bisphosphonate (or denosumab) treatment can be said to be safe, or the magnitude of the risk understood.

A number of the heterozygous cases in Table 2 had received bisphosphonate treatment, but the limited information available suggests the risks of fracture are substantially lower than in those with bi‐allelic mutations. Teriparatide has been used as a treatment for HPP patients with femoral fractures, with varying degrees of success.^6^, ^15^, ^17^, ^18^, ^19^, ^20^ However, in the context of osteoporosis and fractures, teriparatide treatment is limited to 18 months and its effects on bone density wane thereafter, so antiresorptive treatment is recommended after its use.^33^ This, then, does not avoid the dilemma of whether it is safe to use bisphosphonates in adults with HPP and a single heterozygous mutation. In HPP, teriparatide's effects are also short‐lived.^15^ Klidaras and colleagues used short‐term treatment with asfotase alfa, the novel enzyme replacement therapy approved for infantile, childhood and juvenile HPP, in 2 adult subjects with bi‐allelic mutations, with good effect on fracture healing,^23^ but this treatment is unlikely to be widely available for adults.^34^

There is clearly still much to learn about adult HPP, in particular how to manage the frequently encountered patients with osteoporosis alongside mild or asymptomatic HPP with a single *ALPL* mutation. There are insufficient data to state definitively that bisphosphonates are contraindicated in such circumstances.

## Disclosures

All authors state that they have no conflicts of interest.
